# Withdrawal of treatment in a pediatric intensive care unit at a Children’s Hospital in China: a 10-year retrospective study

**DOI:** 10.1186/s12910-020-00517-y

**Published:** 2020-08-12

**Authors:** Huaqing Liu, Dongni Su, Xubei Guo, Yunhong Dai, Xingqiang Dong, Qiujiao Zhu, Zhenjiang Bai, Ying Li, Shuiyan Wu

**Affiliations:** 1 Health Supervision Institute of Gusu District, Suzhou, 215000 Jiangsu China; 2 Department of Intensive Care Unit, Children’s Hospital of Soochow University, No.92, Zhongnan street, Suzhou Industrial Park, Suzhou, Jiangsu China

**Keywords:** Withdrawing treatment, Premature withdrawal, Children, Pediatric intensive care unit

## Abstract

**Background:**

Published data and practice recommendations on end-of-life care generally reflect Western practice frameworks; there are limited data on withdrawal of treatment for children in China.

**Methods:**

Withdrawal of treatment for children in the pediatric intensive care unit (PICU) of a regional children’s hospital in eastern China from 2006 to 2017 was studied retrospectively. Withdrawal of treatment was categorized as medical withdrawal or premature withdrawal. The guardian’s self-reported reasons for abandoning the child’s treatment were recorded from 2011.

**Results:**

The incidence of withdrawal of treatment for children in the PICU decreased significantly; for premature withdrawal the 3-year average of 15.1% in 2006–2008 decreased to 1.9% in 2015–2017 (87.4% reduction). The overall incidence of withdrawal of care reduced over the time period, and withdrawal of therapy by guardians was the main contributor to the overall reduction. The median age of children for whom treatment was withdrawn increased from 14.5 months (interquartile range: 4.0–72.0) in 2006 to 40.5 months (interquartile range: 8.0–99.0) in 2017. Among the reasons given by guardians of children whose treatment was withdrawn in 2011–2017, “illness is too severe” ranked first, accounting for 66.3%, followed by “condition has been improved” (20.9%). Only a few guardians ascribed treatment withdrawal to economic reasons.

**Conclusions:**

The frequency of withdrawal of medical therapy has changed over time in this children’s hospital PICU, and parental decision-making has been a large part of the change.

## Background

Whether to withdraw treatment from critically ill children is an inevitable dilemma for parents and physicians. Published data and practice recommendations on end-of-life care generally reflect Western practice frameworks [1, 2]. In regions with different social cultures, religions, ethnicities, health care levels, and economic development, people often have different attitudes towards withdrawal of treatment. Even in the same region, there are controversial opinions regarding withdrawing treatment [1, 3, 4]. China has the largest number of children in the world, with 220 million children aged 0–14 years [5]. The Chinese government implemented the Children’s Development Program of China (2011–2020) in 2011 with the aim of reducing the mortality of infants and the under 5 mortality rate (U5MR) to 10 and 13 per 1000 live births, respectively [6]. Five years after implementation of the program, the interim statistical monitoring report showed that the infant mortality rate and U5MR were reduced by 5 and 5.7 per 1000 infants compared with 2010, respectively [7]. However, the report did not provide statistical data pertaining to withdrawing treatment for critically ill children, which would have contributed to infant mortality and the U5MR.

There are limited data on withdrawing treatment in pediatric intensive care units (PICUs) in China. It was recently reported that deaths of children whose treatment was withdrawn accounted for 68.3% of the total deaths and 7.8% of the total hospitalizations in a PICU at a tertiary hospital in China [8]. Discharge against medical advice (DAMA), also known as self-discharge, was once common in China: a cross-sectional nationwide study in China including children and adults showed that the proportion of self-discharge reached 43% in 2003, although it fell to 30% in 2011 [9]. In general, self-discharge means that all or part of the patient’s treatment is terminated or withdrawn, although not in all cases. The self-discharge rate for children in general pediatrics at a university affiliated hospital in Henan Province, China from 2011 to 2012 was 18.7% [10]. In a tertiary children’s hospital in Australia, the rate of self-discharge was far lower than that reported above in China, at 1.4% between 2011 and 2015 [11]. Rates of self-discharge among pediatric patients in Africa and the Middle East, ranging from 1.5% to over 6% [11], are also very different from those in China. The self-discharge rate for children in China may be substantially different from that in other countries, but this difference is largely due to the different inclusion criteria for self-discharge in the research from China and other locations. For example, a child transferred to another hospital was considered as self-discharge in Zhao’s research, but not for the results of Sealy et al. [10, 11].

There is currently no relevant legislative provision in China for withdrawing treatment [12]. Owing to the special relationship between physicians and patients and the lack of related laws, whether or not to withdraw treatment is often decided by the child’s guardians, although these decisions may not be in the best interest of the child. Key decisions may include whether or not to withdraw antibiotics, infusions, oxygen inhalation and other life-sustaining therapy from children. There are challenges in working at the interface between clinical practice and the law in this highly emotive area [13]. In the past few decades, economic, healthcare, and social conditions in China have changed significantly. To better understand withdrawal of treatment in China, we report on data from a regional children’s hospital in eastern China during recent years.

## Methods

### Study participants

The study was approved by the hospital ethics committee. The study included pediatric patients who were admitted to the PICU of the Children’s Hospital of Soochow University from 2006 to 2017. The hospital is the only tertiary and class-A hospital in Suzhou and the major hospital to admit and treat critically ill children in the region. The inclusion criteria were as follows: (1) children who met PICU admission standards and were treated in the PICU; and (2) children whose treatments were withdrawn. The exclusion criteria were as follows: (1) admission to the PICU, but subsequently transferred to the general wards or departments; (2) > 14 years of age; (3) in a near-death state and treatment was abandoned at the time of admission; and (4) children with brain death.

### Definition of withdrawing treatment

Withdrawing treatment was divided into two categories, defined as follows: (1) medical withdrawal: the child was in a permanent, irreversible coma or death was inevitable, or treatment in a child for whom pursuing treatment was futile, and the guardian requested the medical staff to limit or withdraw life-sustaining treatment; (2) premature withdrawal: also defined as treatment abandonment, with guardian refusal of active treatment of a severely ill child for whom treatment was indicated or for whom there was some chance of survival, including the following situations: (a) the guardian elected to withdraw care and took the child from the hospital against medical advice; and (b) the child was still severely ill and the guardian did not authorize or ask the doctor to cease the treatment that was expected to improve the prognosis. The patient’s condition and prognosis were based on the clinical judgment of the medical team.

### Data collection and reporting of reasons for withdrawal

The age, sex, place of residence, type of disease, length of stay in the PICU, and condition at the time of discharge were collected from the hospital database (data for 2013 and 2014 were not available). Data on the condition of the child and the withdrawn treatment were collected from the “informed consent” and “doctor–patient conversation record” documents. These documents were produced during hospitalization when the child’s condition was serious or worsened, when she or he needed special treatment or examination, or required expensive treatment. The guardian’s self-reported reasons for abandoning the child’s treatment were recorded from 2011. All the guardians who were asked to report the reasons for abandoning signed the informed consent to enter the study.

### Statistical analyses

Age and days in PICU are presented as the median and interquartile range (IQR). A Wilcoxon two-sample test was used for comparison of age and days in PICU between two groups. Categorical variables are presented as the frequency (%), and a chi-square test or Fisher’s exact test was used for intergroup comparisons. A trend test was used for analyzing the change in the incidence of withdrawing treatment across time from 2007 to 2017. SAS 9.3 was used for data processing and statistical analyses. All tests were two-tailed and *p* < 0.05 was considered statistically significant.

## Results

### General characteristics of the children and incidence of withdrawal of treatment

From 2006 to 2017 (excluding 2013 and 2014), a total of 8006 children were admitted to the PICU; treatments were withdrawn from 680 children, including medical withdrawal in 174 children and premature withdrawal in 506 children. The general characteristics of children whose treatment was withdrawn are shown in Table 1. The age of children who experienced medical withdrawal was significantly higher than those who experienced premature withdrawal (median: 24 vs. 8 months, *p* < 0.001). There was a significant difference in primary disease between children who experienced premature withdrawal and medical withdrawal (infectious proportion: 20.7 vs. 35.6%, *p* < 0.001).

**Table 1:** Characteristic of children whose treatment was withdrawn

Variables	Total, *n* = 680	Medical withdrawing, *n* = 174	Premature withdrawing, *n* = 506	p*
Age*, (month)				< 0.001
Median(IRQ)	12.0 (3.0, 48.0)	24.0 (8.0,73.0)	8.00 (3.0,36.0)	
Days in ICU				0.695
Median(IRQ)	2.0 (1.0,7.0)	3.00 (1.0,8.0)	2.00 (1.00,6.0)	
Gender, n(%)				0.380
Boy	395 (58.1)	106 (26.8)	289 (73.2)	
Girl	285 (41.9)	68 (23.9)	217 (76.1)	
Residence, n(%)				< 0.001
Countryside	93 (13.7)	19 (10.9)	74 (14.6)	
Town	244 (35.9)	63 (36.2)	181 (35.8)	
Urban	182 (26.8)	68 (39.1)	114 (22.5)	
Unknown	161(23.7)	24 (13.8)	137 (27.1)	
Primary disease, n(%)				
Non-infectious	464 (68.2)	138 (79.3)	326 (64.4)	< 0.001
Infectious	216 (31.8)	36 (20.7)	180 (35.6)	

* *p* value for medical withdrawing vs. premature withdrawing

**Fig. 1:**
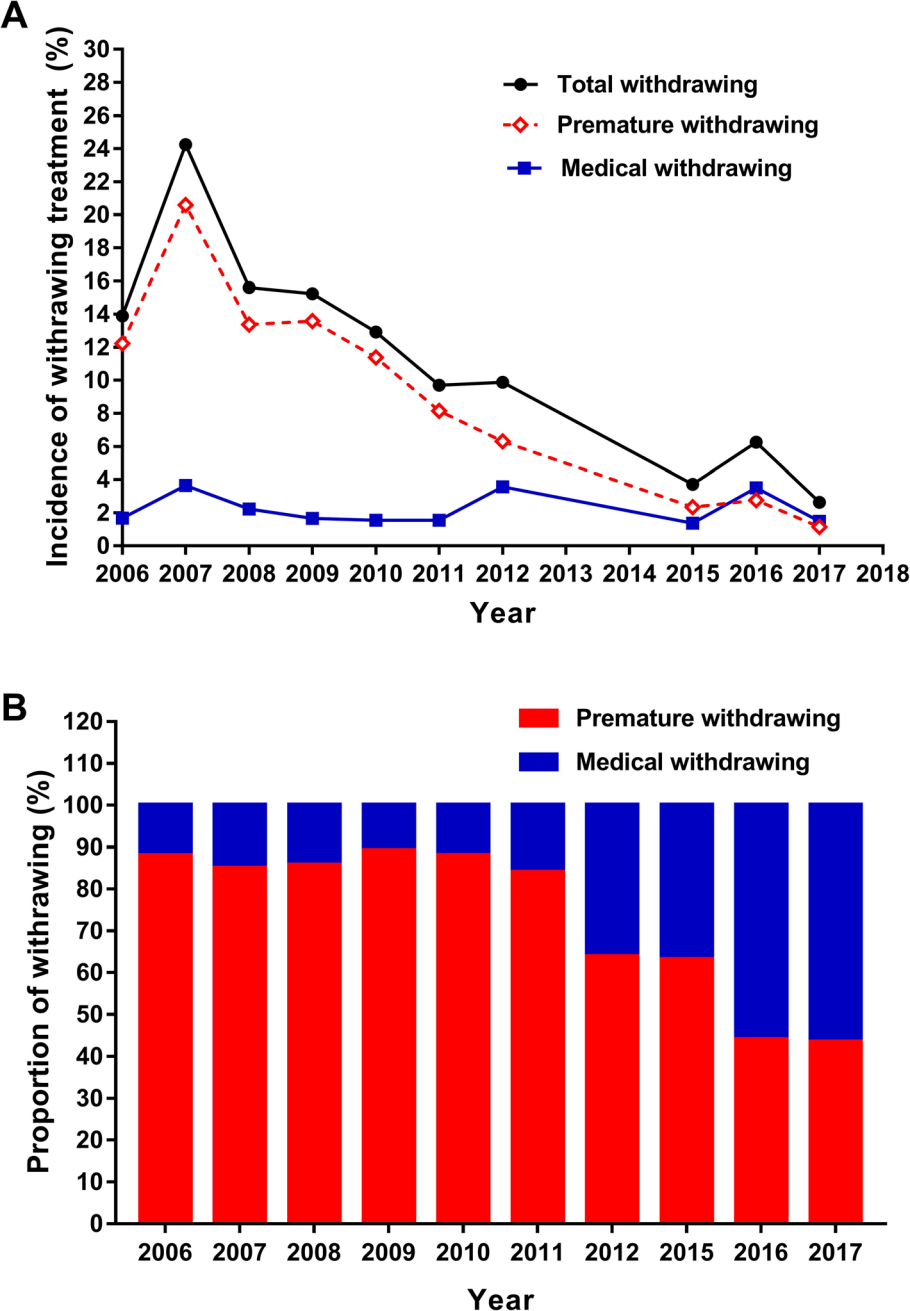
Incidence and proportion of withdrawing treatment in 680 children during 2006–2017

The year-incidence curve is shown in Fig. 1a. The highest incidence of total withdrawal of treatment (24.3%) and the highest incidence of premature withdrawal (20.6%) occurred in 2007. The incidence of total withdrawal of treatment and premature withdrawal followed a year-by-year decreasing trend from 2007 to 2017 (*p* < 0.001). The incidence of total withdrawal of treatment dropped from its highest point (24.3%) in 2007 to its lowest point (2.6%) in 2017; the 3-year average incidence dropped from 17.5% in 2006–2008 to 4.0% in 2015–2017 (a 77.1% reduction). The 3-year average incidence of premature withdrawal dropped from 15.1% in 2006–2008 to 1.9% in 2015–2017 (an 87.4% reduction). There was no significant change in the incidence of medical withdrawal, which was 2.4% in 2006–2008 and 2.1% in 2015–2017. The proportion of premature withdrawal among all children whose treatment was withdrawn declined from 88.0% in 2006 to 43.5% in 2017 (Fig. 1b). The decrease in cases of premature withdrawal contributed most of the decrease in total withdrawal.

**Fig. 2:**
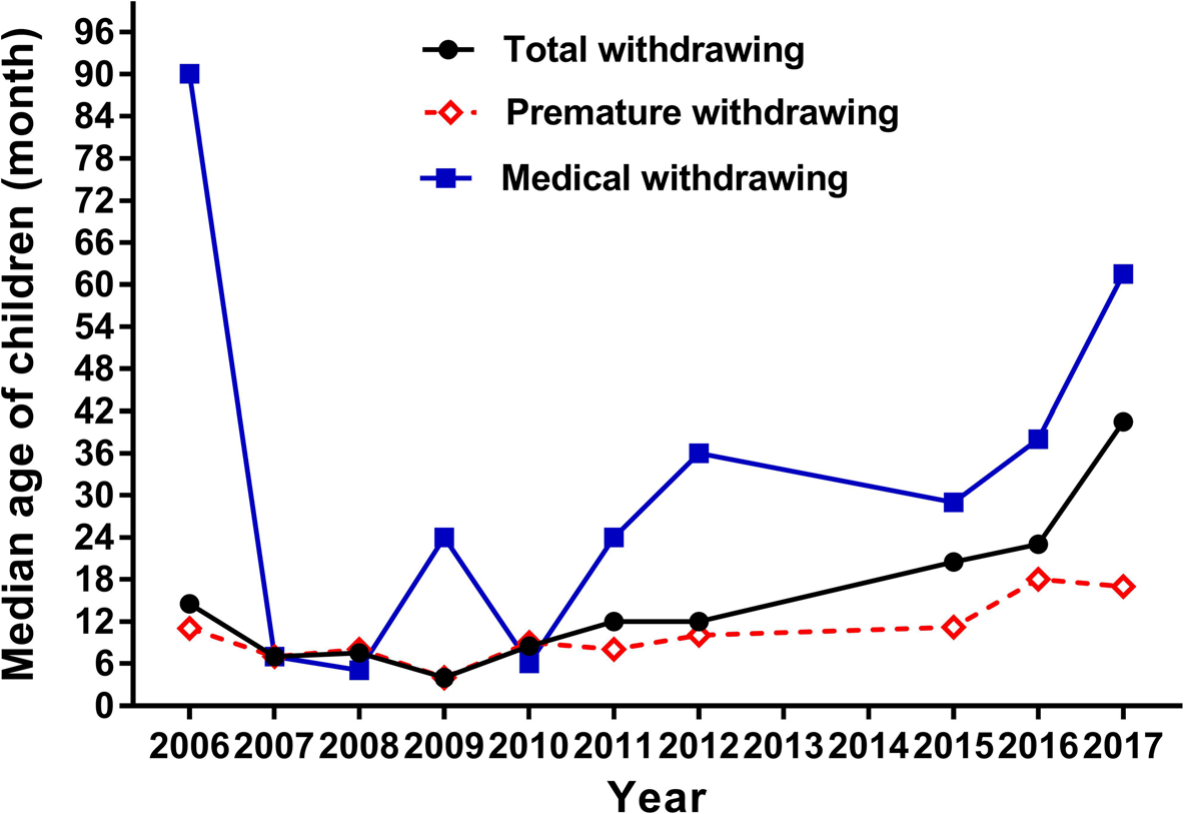
The median age of children whose treatment was withdrawn during 2006–2017

The median age of the children is shown in Fig. 2. There was a downward trend in the median age of all children before 2009, but this increased significantly each year from 2009; the median age increased from 4.0 months (IQR: 2–24) in 2009 to 40.5 months (IQR: 8–99) in 2017. The group who experienced medical withdrawal had a higher increase in age than the group who experienced premature withdrawal.

### Reasons given by guardians for withdrawing treatment and condition of children at the time of discharge

Reasons given by guardians for withdrawing treatment are shown in Table 2. Among the 326 children whose treatments were withdrawn in 2011–2017, “illness is too severe” ranked first, accounting for 66.3%, followed by “condition has been improved” (20.9%). Almost all guardians (96.1%) of children who experienced medical withdrawal self-reported the reason as “illness is too severe”; a few guardians (3.9%) self-reported the reason as “condition has been improved”. For guardians of children who experienced premature withdrawal, these two reasons accounted for 46.7 and 32.0%, respectively. Among all the guardians of children who experienced premature withdrawal, seven (3.5%) self-reported “economic reason” and one (0.5%) self-reported “unclear diagnosis”.

**Table 2:** Reasons given by guardians for withdrawing treatment

Reasons	Total, *n* = 326,(%)	Medical withdrawing, *n* = 129,(%)	Premature withdrawing, *n* = 197,(%)	p*
Illness is too severe	216 (66.3)	124 (96.1)	92 (46.7)	< 0.001
Condition has been improved	68 (20.9)	5 (3.9)	63 (32.0)	
Economic reason	7 (2.1)	0 (0.0)	7 (3.5)	
Unclear diagnosis	1 (0.3)	0 (0.0)	1 (0.5)	
Unstated reason	34 (10.4)	0 (0.0)	34 (17.3)	

* p value for medical withdrawing vs. premature withdrawing

Among the 326 children whose treatments were withdrawn in 2011–2017, 132 (40.5%) died following discharge; among these, 98 deaths followed medical withdrawal and 34 deaths followed premature withdrawal (mortality rate: 76.0% vs 17.3%, *p* < 0.001).

### Treatment modalities that were withdrawn

The life-sustaining treatment modalities that were withdrawn are shown in Table 3. The most frequent modalities withdrawn were intravenous administration, ventilation, and intubation. In 24% of cases, all three treatment modalities were withdrawn.

**Table 3:** Treatments that were in place and then withdrawn from children

Treatments	Total, n = 680,(%)	Medical withdrawing, n = 174,(%)	Premature withdrawing, n = 506,(%)	p*
Intravenous	437 (64.3)	103 (59.2)	334 (66.0)	0.106
Ventilation	279 (41.0)	105 (60.3)	174 (34.4)	< 0.001
Intubation	277 (40.7)	103 (59.2)	174 (34.4)	< 0.001
Antimicrobial therapy	113 (16.6)	19 (10.9)	94 (18.6)	0.019
Inotropic and vasopressors	70 (10.3)	7 (4.0)	63 (12.5)	0.002
Dialysis	25 (3.7)	8 (4.6)	17 (3.4)	0.454
Transfusion of blood products	21 (3.1)	3 (1.7)	18 (3.6)	0.228
Nutrition	9 (1.3)	3 (1.7)	6 (1.2)	0.701

* *p* value for medical withdrawing vs. premature withdrawing

## Discussion

Withdrawing treatment is not only a medical ethical issue but also a social issue. There has been considerable debate about how to implement withdrawal of treatment. Some scholars in China believe that withdrawal of treatment in ICUs should follow the principle of benefit and respect the patient’s willingness and the fairness principle [14]. People also believe that decisions on withholding/withdrawing treatment need to take account of the likely success, benefits, burdens and risks of treatment, as well as the patient’s presumed wishes [4]. Ethicists believe that the best interest standard provides insufficient guidance for decision-making regarding children and does not reflect the actual standard used by medical providers and courts; the harm principle provides a more appropriate threshold for state intervention than the best interest standard [15]. For children, however, withdrawing treatment is decided by their guardian(s) in China, as children do not have full legal capacity, and guardians’ decisions are not always in the best interest of the child. Therefore, in this study we classified cases of withdrawing treatment into two categories: children who were unlikely to survive and whose treatment was withdrawn for medical reasons, and children for whom a treatment was indicated but whose guardian(s) chose to abandon treatment.

For the treatment of children with severe illness in China, the general practice of physicians is to have a conversation with the child’s guardian, introduce the child’s condition to the guardian, provide medical advice, and discuss treatment methods and prognosis, after which the guardians are asked to make a decision. In many cases, even if a child has a chance of survival, their guardians choose to abandon treatment. When this happens, the medical staff will try their best to persuade the guardian not to give up, or will even help the guardian to solve some difficulties. However, unfortunately, there will always be some unexpected disputes, and medical staff even face the risk of legal liability. Therefore, in general, medical staff have to comply with the requirements of the guardian. When patients cannot articulate their wishes in American hospitals, it has been reported that ICU physicians and nurses usually leave final decisions in the hands of the families [16]. Despite extensive experience with critically ill patients and the availability of prognostic scoring systems, prognostication generally remains imprecise in the ICU; physicians cannot say in absolute terms whether a child will die or will experience poor functional outcomes [17], and fear of litigation is a major barrier to informing a child’s guardians properly in Greece [18]. Physicians in China experience similar constraints, which may damage communications and cause resentment. There are official guidelines for withholding and withdrawing therapy for critically ill patients in some countries and regions [1, 19–23].

Researchers believe that several key ethical concepts play a foundational role in guiding end-of-life care, including the distinctions between withholding and withdrawing treatments, between actions of killing and allowing to die, and between consequences that are intended versus those that are merely foreseen [24]. There is no legal procedure or official guideline for withdrawing treatment in China. In China, especially in the past decade, tension and deterioration of the doctor–patient relationship have been increasing, there have been many disputes and contradictions between doctors and patients caused by patients’ treatment choices, and some medical staff have even suffered injuries inflicted by patients or patients’ families. For instance, on October 3, 2016, a pediatrician in Shandong Province was killed by the father of a girl he had treated, and on December 24, 2019, a Beijing emergency physician was killed by a family member of a 95-year-old patient with advanced cancer. In such a situation, in order to avoid the trouble caused by medical disputes, doctors have generally adopted defensive medicine: they will use more obscure technical terms to describe a patient’s condition accurately in the communication process with patients, although these technical terms may not be fully understood by patients and their families [25–28]. In evaluations of the prognosis and treatment of severely ill patients, doctors have become more conservative when discussing options with patients or their families, especially with importunate patients or their families [27, 28]. This makes it difficult for this subset of patients to obtain more active treatment opinions from doctors.

This study showed that over the past decade in the PICU, there has been a decrease in the incidence of withdrawing treatment, which was mainly contributed to by a decline in premature withdrawal. This suggests that guardians are more willing to treat their children actively. The increase in the age of children whose treatment was withdrawn also suggests that guardians are more active in treating their children, although this increase may be due to the increase in the age of children admitted to PICU. It has been reported that guardian withholding or withdrawing of intensive care for extremely preterm infants at the limits of viability has become more acceptable than it was 20 years ago in Germany, Switzerland, and Austria [29]. The proportion of PICU patients from whom life-sustaining treatment was withheld or withdrawn was 1.5% in Chile from 2004 to 2014 [30]. The medical withdrawal defined in our study is equivalent to the withdrawal of life-sustaining treatment mentioned in the above literature. Compared with other countries and regions, the incidence of withdrawing life-sustaining treatment shown in our study in recent years was moderate. The group of children undergoing premature withdrawal of treatment, as defined in this study, mainly comprised children who were discharged against medical advice. Therefore, we speculate that the rate of self-discharge from PICU in our hospital in 2015–2017 was close to that reported for a tertiary pediatric hospital in Australia [11].

Decisions on end-of-life care for neonates shifted from active resuscitation to non-active resuscitation in Korea between 2001 and 2015 [31]. In contrast, the proportion of cases of non-active resuscitation for critically ill children in China is declining. In our opinion, there are several possible reasons for the change in attitude among guardians of critically ill children towards withdrawing treatment, including economic changes, improvements in medical technology, higher education of parents, and a reduction in discrimination against girls. The economic status of children’s families has improved and health insurance covers more residents over the past decade [9]; therefore, families are more capable of paying medical expenses. It is interesting to note that a short economic crisis occurred in China between 2007 and 2008, and the incidence of withdrawing treatment, especially premature withdrawal, reached a peak in 2007. Indeed, economic factors are key in deciding whether or not to abandon treatment [32]. Other studies have also shown that per capita GDP has a high negative correlation with infant mortality in China [33]. The proportion of people with higher education doubled between 2006 and 2017 in China [34], and it has been reported that a low level of education for the father was associated with discharge against medical advice in Iran [35].

In this study, more than half of guardians stated that their reason for withdrawing treatment was that the child’s condition was too severe. Only a few guardians ascribed withdrawing treatment to economic reasons, which is inconsistent with another study in which economic reasons accounted for half of the total [8, 36]. This difference may be due to variations in the study method. Our medical documents only recorded guardians’ self-reported reasons for treatment withdrawal, which may have introduced a bias. Children at the time of withdrawal of treatment had lower disease severity than at admission [36], and one in five guardians cited “condition has been improved” as a reason for withdrawing treatment in this study; most of these were guardians of children who experienced premature withdrawal. We suggest that this was not representative of the true reason for withdrawing treatment, and that guardians may have moderated their statements to alleviate their guilt. Under the influence of Chinese Confucian culture, guardians are used to expressing compromise. When a guardian is asked to report the reasons for withdrawal of treatment, he/she tends to state the apparent objective phenomenon instead of the real reason. We believe that the main reasons for premature withdrawal may be related to economic status and poor and uncertain prognosis; research data from Changsha in China also showed that these are the main reasons [8]. Although China has established a basic medical insurance system covering almost all residents in the past decade [9], insurance coverage of children’s serious illnesses is not perfect, the proportion and amount of out-of-pocket expenses for medical care for serious illnesses are still high, and continuing treatment will impose a heavy economic burden. We observed that premature withdrawal was rare for children raised in social welfare institutes, in large part because the treatment expenses for such children are guaranteed by the government. When the prognosis with a treatment is poor or uncertain, especially for those whose treatment costs a lot of money, and may not ensure survival, guardians who are short of money are more likely to give up the treatment. During our clinical PICU experience, although sometimes doctors definitely tell the guardian that the child can survive after treatment, some guardians are afraid that serious sequelae will affect the quality of life of the family and they decide to give up. This tendency of guardians can also be seen at social welfare institutes in China, where most of the children have been abandoned by their parents because of congenital diseases.

Although death practices are changing in China, the idea of a death occurring at home or in the person’s home town, in the main hall in the presence of ancestor tablets, is still cherished [3]. This may be one of the factors affecting decisions by guardians. The low proportion of deaths in hospital for children whose treatment was withdrawn prematurely and the fact that some children experiencing medical withdrawal survived when discharged from hospital may be influenced by the death culture in China. The mortality rate among children in our study following withdrawal of life-sustaining treatment was significantly lower than that in a PICU in Australia [37]. This is because some children in our study retained limited maintenance measures (e.g., Ambu) after most of their life-sustaining treatments had been removed to allow them to leave the hospital immediately to let the death occur at home or in their home town. Similar practices can be observed elsewhere: home deaths for critically ill babies/children do occur in the UK, albeit infrequently [38].

When interpreting the results from this study, some limitations should be considered. This was a single-center retrospective study. The region where the hospital is located is undergoing rapid urbanization, and is an economically developed region in China. The results of this study are not representative of all regions of China. The impact of culture, healthcare insurance status, religion, and education on the withdrawal of treatment has not been studied.

## Conclusions

The frequency of withdrawal of medical therapy has changed over time in this children’s hospital PICU, and parental decision-making has been a large part of the change.

## Data Availability

The datasets used and/or analysed during the current study are available from the corresponding author (Shuiyan Wu) on reasonable request.

## Abbreviations

PICU: Pediatric intensive care unit
U5MR: Under 5 mortality rate
DAMA: Discharge against medical advice
IQR: Interquartile range
